# Factors of body dissatisfaction among lebanese adolescents: the indirect effect of self-esteem between mental health and body dissatisfaction

**DOI:** 10.1186/s12887-022-03373-4

**Published:** 2022-05-23

**Authors:** Sara Al-Musharaf, Radoslaw Rogoza, Mariam Mhanna, Michel Soufia, Sahar Obeid, Souheil Hallit

**Affiliations:** 1 Department of Community Health Sciences, College of Applied Medical Sciences, King Saud University, Riyadh, Saudi Arabia; 2 Cardinal Stefan Wyszyński University, Warsaw, Poland; 3 Social Innovation Chair, University of Lleida, Lleida, Spain; 4 School of Medicine and Medical Sciences, Holy Spirit University of Kaslik, P.O. Box 446, Jounieh, Lebanon; 5 Social and Education Sciences Department, School of Arts and Sciences, Lebanese American University, Jbeil, Lebanon; 6 Psychology Department, College of Humanities, Effat University, 21478 Jeddah, Saudi Arabia; 7 Research Department, Psychiatric Hospital of the Cross, Jal Eddib, Lebanon

**Keywords:** Body dissatisfaction, Adolescents, Lebanon, Self-esteem, Depression

## Abstract

**Background:**

Body dissatisfaction (BD) rates are alarmingly high, especially among adolescents, thus. Having a better understanding of correlates associated with BD seems to be an important issue in this developmental context. Furthermore, as adolescence is an essential time in the development of self-perception and self-esteem the current study assesses factors associated with BD among Lebanese adolescents and evaluates the indirect effect of self-esteem between depression/anxiety/stress and BD.

**Methods:**

This is a cross-sectional study among 555 Lebanese adolescents, ages 15–18, who completed an online survey incorporating BD, socioeconomic status, weight and height, physical activity index, Rosenberg Self Esteem Scale, Beirut Distress Scale, Hamilton Anxiety Rating Scale, Patient Health Questionnaire-9, Pittsburgh Sleep Quality Index and Global Physical Activity Questionnaire.

**Results:**

The results of a stepwise linear regression, taking the body dissatisfaction score as the dependent variable, showed that higher BMI (B = 0.47), feeling pressured by media/TV to lose weight (Beta = 2.80), higher depression (Beta = 0.39), exercising to lose weight (Beta = 1.84) and following a diet to lose weight (Beta = 1.58) were significantly associated with more body dissatisfaction, whereas higher self-esteem (Beta=-0.11) and more psychological distress (Beta=-0.21) were significantly associated with less body dissatisfaction. Self-esteem played an indirect role in the associations between anxiety and body dissatisfaction and stress and body dissatisfaction.

**Conclusions:**

BD is common among young Lebanese adolescents. Treating adolescents with low self-esteem and psychological issues is crucial in preventing anticipated BD and future eating disorders.

## Introduction

Body dissatisfaction (BD) is the negative thought, feeling and perception of a person’s physical appearance [1] and how actions towards their own body would greatly influence their health at any phase of their lives, given the “developmental significance” of their body image status [2]. Adolescence is one of the most pivotal phases in the development of self-perception [3]. During the many changes of the pubertal storm an unhealthy, mostly negative, and inaccurate body image can be formed [3].

In the past forty years, BD has been of growing interest in scientific literature [3, 4]. The high prevalence of eating disorders among adolescents compared with the general population has been observed in previous studies [3, 4]. Studies show that almost 60% of adolescents might have eating disorders or are at high risk to develop them [5]. The constant rise in eating disorders, weight status, physical inactivity and the consequent associated health repercussions parallel the rising importance of BD contribution to health [3]. The highest levels of BD are seen in adolescence, early adulthood, and women [6, 7]. Among adolescents, BD is a serious public health problem as it generates a series of health complications including compromised emotional well-being, low self-esteem, depression and eating disorders [8–11]. Additionally, mental status is one of the ten principal causes of health conditions among young women [12].

The prevalence of BD is growing at alarming rates both globally [13–15] and regionally [5, 16–18]. A recent study among 308 Emirati University students between 18 and 25 years old showed that 81% of them had BD [16]. A study of Lebanese high school students found that 60% of boys and 70% of girls were dissatisfied with their bodies [18]. Discrepancies were found between their actual weight and their ideal weight. 44% of the boys aspired to be larger, and 59.5% of the girls desired to be smaller [18]. Hence, these significant rates of regional and Lebanese adolescents with BD would be crucial for assessment and exploring all influencing correlates.

Many factors may play a role in developing BD. Physical, cultural, socio-economics, psychological and social factors [3, 19], along with family and peer influence [20], parental attitude [21], and appearance-specific media pressures [22] influence the body image of people between 12 and 18 years old [3]. The globalization of unrealistic ideal body image among adolescents have driven them to endure overall body image distortion as well as BD dissatisfaction [23]. Even health initiatives that tend to focus on obesity prevention without taking into consideration the body image perception have unintentionally reinforced BD among adolescents, predisposing them to severe eating disorders [24]. Additionally, higher body mass index (BMI) [5, 16, 25–27], younger age [17, 26] and female sex [16, 26] may influence BD among adolescents.

Thus, dissatisfied adolescents may induce unhealthy weight control practices including dieting [28], exercising [16], daily weigh-ins [29], taking diet pills and steroid use [30]. Furthermore, the individual’s psychological status has a role in BD including depression [8, 27], stress [31], anxiety [10] and self-esteem [32–34]. Hence, the psychosocial status may increase the risk of BD, and some studies showed a bidirectional association between them [8, 35, 36]. Political and financial crisis that Lebanese are living, propose the Lebanese adolescents to altered mental health [37], and hence prone to BD.

Multiple studies have shown that self-esteem is associated with BD [32–34]. Adolescence plays a critical role in the development of self-esteem which reflects a person’s self-confidence [3]. Self-esteem has garnered attention in the last decade in that it may be the buffering variable that leads to BD. For instance, self-esteem alter depression, anxiety, and stress [31]. Some studies had been done on Lebanese adolescents and adults concerning BD [18, 38].

Limited studies were found to assess the mediating effect of self-esteem on psychological-BD status. In this study, we strive to replicate those findings by considering multiple psychological factors among Lebanese adolescents, which we believe are more prone to altered psychological status [37]. The majority of adolescents have not received treatment BD and one of the main reasons for that is a lack of a perceived need [39]. Hence, the aim of the present study is to assess factors associated with BD among Lebanese adolescents and evaluate the mediating effect of self-esteem between depression/anxiety/stress and BD. Timely prevention of BD and disordered eating among adolescents is a major public health priority that will reduce multiple health complications later in their lives [4, 37, 39].

## Methods

### Study design

This was a cross-sectional designed study, conducted between May and June 2020, using a snowball sampling technique from all Lebanese governorates (Beirut, Mount Lebanon, North, South, and Bekaa). A soft copy of the questionnaire was created using Google forms, and an online approach was used for the data collection. No credits were given for participation.

### Participants

A total of 555 adolescents currently residing in Lebanon (15 to 18 years old) participated in this study.

#### Minimal sample size calculation

According to the G-power software, and based on an effect size f^2^ = 2%, an alpha error of 5%, a power of 80%, and considering the 16 factors to be entered in the multivariable analysis, the results showed a minimum of 395 participants were needed.

### Data collection

The questionnaire was conceived in Arabic, native language in Lebanon, using already validated scales in the Lebanese adolescents’ population for depression and anxiety, as well as a forward and back translation procedure for other questionnaires including body dissatisfaction scale and self-esteem scale.

The first part of the questionnaire assessed socio-demographic details (age, sex, residency governorate, height, weight, etc.). The body mass index (BMI) was consequently calculated as per the World Health Organization [40]. The household crowding index (HCI), reflecting the socioeconomic status (SES) of the family, was calculated by dividing the number of persons living in the house by the number of rooms in the house; higher HCI reflected a lower SES [41]. The physical activity index was calculated by multiplying the physical activity frequency by its duration by its strength [42].

The second part of the questionnaire constituted the following measures and scales:

#### Body dissatisfaction scale

This study used a forward and back translation procedure to validate the Arabic version of the Body Dissatisfaction subscale out of Eating Disorders Inventory-2 (explained below). This nine-item scale assess the levels of dissatisfaction with the overall shape and particular parts of the body [43]. It was measured on a four-point Likert-type scale, ranging from 0 (*never*) to 3 (*always*). The total score was calculated by the sum of the nine items. The BD total score was divided by the median to show the high and low BD. The higher the score, the greater the body dissatisfaction (Cronbach’s alpha in this study = 0.81).

#### Rosenberg Self Esteem Scale (RSES)

The Rosenberg Self Esteem scale is a self-reported ten-item questionnaire used to assess beliefs and attitudes regarding self-worth [44]. This study used the forward and back translation procedure to use the Arabic version for RSES (explained below). The answers were graded using a four-point Likert-type scale, with answers ranging from 1 (*strongly disagree*) to 4 (*strongly agree*). Higher scores indicated higher self-esteem (Cronbach’s alpha in this study = 0.74).

#### Hamilton anxiety rating scale (HAM‑A)

The Hamilton Anxiety Rating Scale is a widely used instrument in clinics and research for measuring the severity of anxiety symptoms [45]. The scale was translated to Arabic and linguistically validated in the Lebanese population [46]. It comprises fourteen items targeting both psychological and somatic symptom-defined elements. Each question scored on a basic numeric scoring of 0 (*not present*) to 4 (*severe*), with a total score ranging from 0 to 56, with higher scores delineating higher anxiety (Cronbach’s alpha in this study = 0.89).

#### Patient health questionnaire (PHQ-9)

The Patient Health Questionnaire is a nine-item instrument [47] developed for making Diagnostic and Statistical Manual of Mental Disorders-4th Edition criteria-based diagnoses of depressive disorders encountered in primary care and is validated to Arabic language among Lebanese populations [48]. Questions are about the level of interest in doing things, feeling down or depressed, difficulty with sleeping, energy levels, eating habits, self-perception, ability to concentrate, speed of functioning and thoughts of suicide. Each item is scored on a four-point Likert scale, ranging from 0 (*not at all*) to 3 (*nearly every day*). Total scores range from 0 to 27, with higher scores indicating greater depression (Cronbach’s alpha in this study = 0.84).

#### Beirut Distress Scale (BDS-10)

The Beirut Distress Scale, developed in Lebanon [49], was used to assess the intensity of psychological distress by using ten questions. The points range from 0 (*never*) to 3 (*always*), with higher scores indicating higher psychological distress (Cronbach’s alpha in this study = 0.82).

The last part of the questionnaire included general questions retrieved from a previous study [50] about losing weight, dieting, food, external pressures to go on a diet, abuse and family history of eating disorders (e.g. “Do you often hear comments about your weight?”, “Do your relatives comment on your weight?”, “Do you feel pressured to go on a diet?”, “Have you felt pressured by the media to change your diet?”, “Have you followed any diet to lose weight?”). These variables were classified as categorical variables (yes/no answers).

### Forward and back translation

Two bilingual psychologists accomplished the forward and back translations of the body dissatisfaction and self-esteem scales. The original and translated English versions were compared by a psychiatrist for discrepancies, which were resolved by consensus [51–55].

### Statistical analysis

No missing values were found since all questions were forced answers. All the SPSS software version 23 was used to conduct data analysis. The normality of distribution of the body dissatisfaction score was confirmed via a calculation of the skewness and kurtosis; values for asymmetry and kurtosis between − 1 and + 1 are considered acceptable to prove normal univariate distribution [56]. These conditions consolidate the assumptions of normality in samples larger than 300 [57]. The Student t test was used to compare two means respectively. Effect sizes were calculated; According to Cohen, d = 0.2 would be considered as a small effect size, 0.5 represents a ‘medium’ effect size and 0.8 a ‘large’ effect size. The Pearson correlation was used to check for an association between continuous variables. Bonferroni correction was also applied since multiple testing (16 variables) was done; the corrected p-value was 0.05/16 = 0.003. Stepwise linear regression was conducted, taking the body dissatisfaction score as the dependent variable.

The PROCESS SPSS Macro version 3.4, model four [58] was used to calculate three pathways. Pathway A determined the regression coefficient for the effect of depression/anxiety/stress and self-esteem, Pathway B examined the association between self-esteem and body dissatisfaction, independent of psychological illness, and Pathway C estimated the total and direct effect of each psychological illness on body dissatisfaction. Pathway AB calculated the indirect intervention effects. The significance of the indirect effect was determined if the macro generated bias-corrected bootstrapped 95% confidence intervals did not include zero [58]. All variables that showed a significant association in the bivariate analysis according to the Bonferroni correction were taken as independent variables in the final models. Significance was set at a *p* < 0.05.

## Results

### General characteristics

A total of 555 Lebanese adolescents enrolled in this study, with a mean age of 16.66 ± 1.00 years (75.7% females). More details about the students can be found in Table 1. The mean body dissatisfaction score was 12.22 ± 6.51 (median = 13; minimum = 0; maximum = 27; skewness = 0.006; kurtosis=-0.632). According to the median, 277 (45.1%) participants had high body dissatisfaction.

**Table 1:** Sociodemographic and other characteristics of the participants (*N* = 555)

Variable	*N* (%)
**Sex**	
Male	135 (24.3%)
Female	420 (75.7%)
**District**	
Beirut	62 (11.2%)
Mount Lebanon	332 (59.8%)
North	92 (16.6%)
South	29 (5.2%)
Bekaa	40 (7.2%)
	**Mean ± SD**
Age (in years)	16.66 ± 1.00
Household crowding index	0.99 ± 0.52

Categorical variables were presented in percentages and continuous normal distribution variables presented as mean ± standard deviation

### Factors of body dissatisfaction scale

#### Bivariate analysis

A higher mean body dissatisfaction score was seen in females compared to males. Also, participants who weigh themselves daily, diet, exercise, vomit, take medications, starve themselves, and feel pressured by media/TV to lose weight had a higher body dissatisfaction score compared to those who those did not follow these habits (Table 2).

Furthermore, higher body mass index, higher stress, anxiety and depression were associated with more body dissatisfaction, whereas higher self-esteem was significantly associated with lower body dissatisfaction (Table 3).

**Table 2:** Bivariate analysis of categorical variables associated with the body dissatisfaction score

Variable	Body dissatisfaction (mean ± SD)	*p*	Effect size (d)
**Sex**		0.004	0.243
Male	11.00 ± 5.65		
Female	12.81 ± 6.60		
**Diet to lose weight**		**< 0.001**	0.730
No	10.63 ± 6.09		
Yes	15.16 ± 5.94		
**Exercise to lose weight**		**< 0.001**	0.580
No	9.72 ± 6.15		
Yes	13.58 ± 6.18		
**Vomit to lose weight**		**< 0.001**	0.300
No	12.01 ± 6.49		
Yes	14.90 ± 5.30		
**Medications to lose weight**		0.005	0.209
No	12.16 ± 6.49		
Yes	14.47 ± 5.37		
**Starve yourself to lose weight**		**< 0.001**	0.576
No	11.37 ± 6.25		
Yes	15.52 ± 5.92		
**Weigh yourself daily**		**< 0.001**	0.382
No	11.69 ± 6.49		
Yes	14.30 ± 5.83		
**Experience social isolation**		0.317	0.084
No	12.18 ± 6.51		
Yes	12.77 ± 6.23		
**Feeling pressured by media/TV to lose weight**		**< 0.001**	0.866
** No**	10.50 ± 6.08		
Yes	15.73 ± 5.61		

Numbers in bold indicate significant associations with body dissatisfaction according to the Bonferroni corrected *p*-value (*p* = 0.003)

**Table 3:** Correlation of continuous variables with the body dissatisfaction score

Variable	r	*p*
Self-esteem	-0.247	**< 0.001**
Anxiety	0.172	**< 0.001**
Depression	0.327	**< 0.001**
Psychological distress	0.159	**< 0.001**
Physical activity index	0.012	0.772
Household crowding index	-0.091	0.032
Age	-0.005	0.909
Body Mass Index	0.412	**< 0.001**

Numbers refer to Pearson correlation coefficients; those in bold indicate significant associations with body dissatisfaction according to the Bonferroni corrected *p*-value (*p* = 0.003)

### Multivariable analysis

The results of a stepwise linear regression, taking the body dissatisfaction score as the dependent variable, showed that higher BMI (B = 0.47), feeling pressured by media/TV to lose weight (Beta = 2.80), higher depression (Beta = 0.39), exercising to lose weight (Beta = 1.84) and following a diet to lose weight (Beta = 1.58) were significantly associated with more body dissatisfaction, whereas higher self-esteem (Beta=-0.11) and more psychological distress (Beta=-0.21) were significantly associated with less body dissatisfaction (Table 4).

**Table 4:** Multivariable analysis: Stepwise linear regression taking the body dissatisfaction score as the dependent variable

Variable	Beta	β	*p*	95% CI
Body Mass Index	0.47	0.29	< 0.001	0.36–0.58
Feeling pressured by media/TV to lose weight (yes vs. no^a^)	2.80	0.21	< 0.001	1.82–3.78
Depression	0.39	0.34	< 0.001	0.28–0.51
Exercise to lose weight (yes vs. no^a^)	1.84	0.13	< 0.001	0.86–2.81
Psychological distress	-0.21	-0.18	< 0.001	-0.31- -0.10
Diet to lose weight (yes vs. no^a^)	1.58	0.12	0.002	0.60–2.57
Self-esteem	-0.11	-0.10	0.010	-0.20- -0.03

^a^Reference group; *Beta *Unstandardized Beta, *β *Standardized Beta, *CI *Confidence Interval

### Indirect effect of self-esteem

**Fig. 1:**
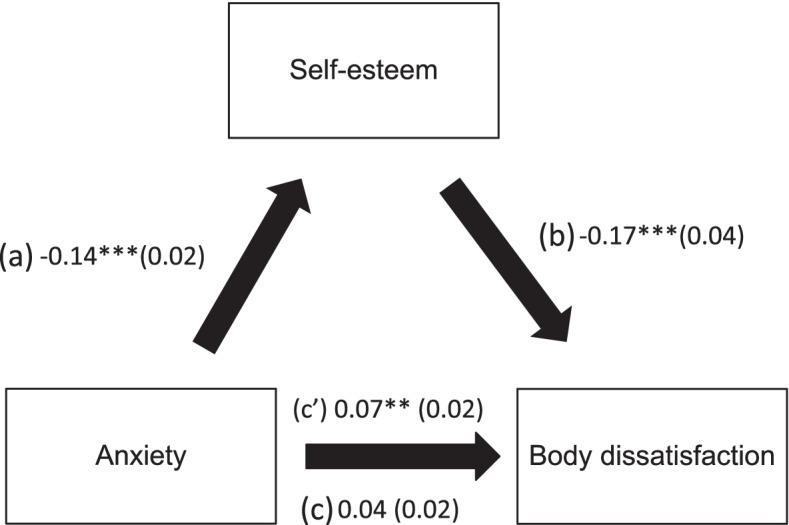
**a** Relation between anxiety and self-esteem; **b** Relation between self-esteem and body dissatisfaction; **c** total effect of anxiety on body dissatisfaction; (**c’**) direct effect of anxiety on body dissatisfaction. Numbers are displayed as regression coefficients (standard error). *p < 0.05; **p < 0.01; ***p < 0.001

**Fig. 2:**
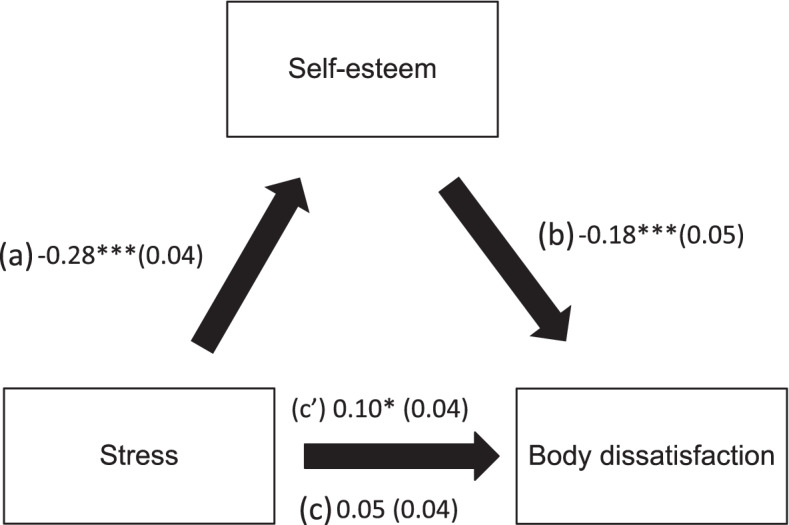
**a** Relation between stress and self-esteem; **b** Relation between self-esteem and body dissatisfaction; **c** total effect of stress on body dissatisfaction; **c’** direct effect of stress on body dissatisfaction. Numbers are displayed as regression coefficients (standard error). **p < 0.01; ***p < 0.001

Self-esteem played an indirect role in the associations between anxiety and body dissatisfaction and stress and body dissatisfaction after adjustment over the following variables: BMI, feeling pressured by media/TV to lose weight, exercise to lose weight, diet to lose weight and experience social isolation (Table 5; Figs. 1 and 2).

**Table 5:** Mediation analysis: Direct and indirect effects of the associations between mental health issues, self-esteem and body dissatisfaction

Independent variable	Direct effect			Indirect effect		
	**Effect**	**SE**	**p**	**Effect**	**SE**	**95% BCa**
Depression	0.29	0.05	< 0.001	0.03	0.02	-0.002-0.07
Anxiety	0.04	0.02	0.055	0.02	0.01	0.01–0.04^a^
Stress	0.05	0.04	0.242	0.05	0.01	0.02–0.08^a^

^a^indicates significant indirect effect

## Discussion

To the best of our knowledge, this is the first study to assess the mediating role of self-esteem in the association between psychological health issues and BD. Greater depression, higher BMI, feeling pressured by media/TV, following a diet to lose weight, exercising to lose weight and daily weigh-ins were significantly associated with higher increase of BD. Higher self-esteem was associated with less BD. Self-esteem had an indirect effect in the associations between depression, anxiety, stress and BD.

### Prevalence

45% of the adolescents in our sample experienced BD. This high prevalence was expected as similar and higher results were found in regional studies; in United Arab Emirates, 36.7% among adolescents [5] and 81% among university students [16]. In Lebanon, an earlier study showed 60% of high school boys and 70% of high school girls had BD [18]. Alarmingly high percentages were also found in India [13], Korea [14], and Brazil [38] indicating that early intervention may be needed to curb its spread. Although congruent with other studies and useful in everyday practice, these results should be interpreted with caution as neither self-report measure alone is able to comprehensive and reliable diagnose any disorder.

### Factors associated with body dissatisfaction

#### Psychological status

##### Depression

In our study, depression is among the independent strongest factors associated with BD. The greater the depression, the more BD among adolescents, in accordance with previous studies among adolescents and early adulthood in different countries [8, 27, 59, 60]. In 2019, a study among 5,888 undergraduates from Egypt, Palestine and Finland showed that BD is associated with depression and that depressive symptoms mediated the observed associations between self-rated health and BD [27]. Also, a Brazilian study of 2,162 older adolescents showed a significant association between dissatisfaction due to being overweight and symptoms of depression after adjusting for sex and nutritional status, and did not find any association with dissatisfaction due to thinness [59]. Additionally, a longitudinal study published in 2020 of 3,753 British adolescents showed that BD in fourteen year olds independently predicts the occurrence of depressive symptoms in eighteen year olds [8]. Another longitudinal study showed that BD in adolescents is a risk factor for depression in early adulthood [60]. It is worth mentioning that BD may increase the risk of depression and people with depression are more susceptible to BD.

The link between body dissatisfaction and mental health is complex. Possible explanations include that adolescents may have negative perceptions of their body image as they want to reach unachievable beauty standards. This can lead to unsuccessful efforts to change their bodies resulting in frustration and depression [61, 62]. Additionally, BD-depression links are supported by neurobiological studies where hypothalamic pituitary-adrenal axis and serotonin system low levels are incorporated in mood disorders and in weight regulation, and brain areas involved in hedonic regulation may play a role in BD and depression [62, 63]. On the other hand, weight gain, which is often observed in patients who are depressed, can lead to BD [35, 36].

##### Psychological distress

In this study, we found that psychological distress had an independent negative association with BD, i.e. less psychological distress among adolescents was associated with higher BD. In contrast to our study, studies on undergraduate women have shown that higher stress was associated with higher BD [64, 65]. A study involving 515 adolescents ages 12–16 found a significant association between higher BD and higher ratings of peer stress and lower self-esteem; these findings suggest that adolescent stress relates to satisfaction with the body and that this stress is specifically focused on the peer environment for both sexes during adolescence [64]. Furthermore, a more recent study done during COVID-19 showed that COVID-19-related stress and anxiety are associated with more BD, which is explained by perceived stress, stressful life events, and trait anxiety [66]. These studies proposed that stress may be linked with greater frequency of negative body thoughts that lead to BD [67]. The controversy in our results may be due to different reasons, the first is that our study assessed psychological distress rather than general stress. Second, maybe these adolescents are less focused on their appearance if they had higher grade point averages. A study indicated that women with higher grade point averages and women interested in full-time professional employment may be less obsessed with their physical appearance [68]. Also, these adolescents with higher academic goals are more stressed [69]. Therefore, they concentrate less on their appearance and have less BD. However, we did not measure the academic performance to prove our theory. Finally, maybe Lebanese adolescents who are more worried about the financial and political problems are having higher psychological distress and are more focused on the event that is causing the stress to try to solve it, therefore, less interested about their appearance, hence less BD.

##### Self-esteem

In this study, self-esteem showed an independent negative association with BD, meaning among Lebanese adolescents lower self-esteem is associated with higher BD. Consistent with earlier scholarly studies, some found similar results among adolescents [32–34, 70]. Macêdo Uchôa et al. studied 1,011 Brazilian students and concluded that adolescents with BD had 5.79 times lower self-esteem in comparisons to adolescents without BD attending public schools (CI 95% 2.06–16.26) and 2.96 times higher in adolescents attending private schools (CI 95%, 1.79–4.88) [33]. Furthermore, a longitudinal study that followed 440 children for five years, after controlling for confounders, showed BD is a risk factor for depressive mood and low self-esteem in both girls and boys but in different phases of adolescence [32]. The above studies, along with ours, revealed a bidirectional association between self-esteem and BD. A main factor of shaping adolescents’ identity is their physical appearance and body image. Very few studies have evaluated the mediating role of self-esteem in the BD-psychological status relation.

To the best of our knowledge, this the first study in the Middle East that assess the mediating role of self-esteem on the relationship between BD and psychological status i.e. depression, anxiety and stress among adolescents. This study found that self-esteem mediated the association between BD and depression by 12.41%, while there was no mediation effect for self-esteem with anxiety and BD. Only three studies were found among adolescents [10, 11, 19]. Duchesne et al. conducted a study of 409 Canadian adolescents fourteen to eighteen years old and they revealed a significant indirect effect for self-esteem for both anxiety and depression with BD, confirming the mediating role of self-esteem [11]. Depression lowers self-esteem which in turn increases BD. Koronczai et al. also showed that self-esteem partially mediated the link between anxiety and BD and fully mediated the link between depression and BD [11]. Choi et al. examined adolescents from the US and Korea and showed that self-esteem mediated the association between BD and depression among adolescents. There was a greater effect on the Americans than the Koreans, implicating cultural differences [19].

Self-esteem had an indirect effect in the associations between anxiety, stress and BD. Currently, only one study has been found to assess self-esteem meditation of stress and BD among adolescents [31]. Murray et al. studied 298 adolescents and the association between stress and BD one year apart. Using a prospective manner revealed that the relationship was unable to account for stress and BD over time, and that other variables i.e. self-esteem explained the variation over time [31]. In other words, these findings suggest that mental health issues may indirectly contribute to increased adolescents’ BD through low self-esteem. This shows the importance of self-esteem established during adolescence [71] and focusing on its treatment will be more effective. This can be accomplished by addressing all factors that may influence its healthy development of self-esteem during adolescence including peer and social influence, parental influence, mental health, and well-being. Focusing on self-esteem may lower future BD and worsen psychological status and eating disorders [72].

### Other factors

Like many other studies, ours found that BMI was among the strongest independent factors associated with BD [25] [5, 16, 26, 27, 73]. These results reveal the significance of BMI in determining the actual body weight for overweight and obese adolescents [74]. Furthermore, this study showed an independent association between BD and the feeling of media/TV’s pressure to lose weight. Several studies are consistent with the current adolescent studies in Ghana [57], Malaysia [75], Jordan [17], Korea [76] Italy and France [77]. A study that took 299 girls (average age 19.9 years) and exposed them to false advertisements found that adolescents with initial BD reported higher BD after being exposed to images of ideally thin models over images of average-size models [77]. The media plays a vital role in formulating what is attractive in society. Media messages reflect adolescents’ beliefs regarding physical appearance [78]. This is of great concern in Lebanon, as it is the Middle East’s fashion and modelling center [79]. Efforts should be made to change the “image of the individuals” in media, particularly regarding using body images in advertisements.

Other lifestyle patterns related to BD were diet, exercising to lose weight and daily weigh-ins. This is clinically significant because negative body image among adolescents leads to strict dieting, unhealthy eating habits and excessive exercise. Nonetheless, adolescents who engaged in weight control practices could be disappointed with the outcome if their expectations to achieve societal beauty standards are not met [80], leading to BD. These weight control practices were similar to previous studies [15, 16, 28, 29].With respect to sex, Radwan et al. conducted a study on Emirati University students proposed that female students who wanted to be thinner preferred following a diet for weight loss, whereas males who desired to be heavier were physically active and less likely to follow a diet [16].

### Limitations

This study acknowledged several limitations. First, the self-reported data may be affected by adolescents’ subjective responses and recall bias. The second limitation was it is cross-sectional study, thus limits causality. While most mediation studies have used a cross-sectional design, a longitudinal study would shed light on the association between the variables studied (BD, self-esteem, depression, stress and anxiety). Third, the majority of the sample were females, thus we couldn’t analyse the data based on sex. Our study did not show any significant difference between Lebanese girls and boys. Girls showed a trend that disappeared in the multivariate regression. This may be due to the small sample size of boys. Residual confounding bias might be possible since not all factors associated with BD were considered in this paper. Body dissatisfaction is multifactorial however, the method to evaluate it does not contemplate the different components. Finally, it was difficult to compare the results of our study to previous studies due to different tools, methodologies and cultures. BMI was based on participants’ self-report and not actual measurements. The Cronbach’s alpha of the body dissatisfaction subscale is suboptimal. Finally, the data collection was performed between May and June 2020, which corresponds to the period of first wave of COVID-19 pandemic, being a critical period regarding mental health of worldwide people, including adolescents.

This study’s main contribution is that it was the first study in the Middle East to assess the mediating effect of self-esteem on BD and physiological status including depression, stress and anxiety among adolescents. It integrated different factors and their association with BD among a single ethnic group of older adolescents. The majority of studies were done on young adults [5, 16, 73, 76] while others were done on the entire adolescent period (10–19 years old) [5, 13] or early adolescence [26, 81], few studies were done on older adolescents [25, 33, 70]. The third contribution was the use of BD subscale of EDI-2, as the majority of studies used the stunkard figure rating scale silhouette [10, 16, 26, 73] which the arrangement of figure may cause reporting bias toward thinner Fig. [82]. The figure rating scale silhouette has been used worldwide to screen for eating disorders in the general population including BD, as well as in clinical evaluations [83]. Furthermore, it has been tested for its validity and reliability among this sample.

## Conclusions

In conclusion, BD is prevalent locally and globally, and assessing its correlates is of utmost importance. We must protect self-esteem during adolescence to reduce the risk of future eating disorders [72]. This study concluded that BMI and depression were both high correlates to BD along with media influence, self-esteem, stress and weight control practices including dieting, exercising and daily weigh-ins. Self-esteem was a buffer between depression and stress associations with BD. Focusing on self-esteem among adolescents reduces side effects including BD and worsening psychological statuses that often carry over into early adulthood. Future longitudinal studies should be carried out in at-risk populations, examining how physical, cultural and social groups, as well as media and health factors affect youths’ self-esteem. Additionally, we recommend studying all potential health awareness programs that can improve adolescent awareness about the importance of healthy practices in daily life, their body image, real weight, weight status and self-esteem.

## Data Availability

The datasets generated and/or analysed during the current study are not publicly available due their institutions policies but are available from the corresponding author (SH) on reasonable request.

## Abbreviations

BD: Body dissatisfaction
BMI: body mass index
HCI: household crowding index
SES: socioeconomic status
RSES: Rosenberg Self Esteem Scale
HAM‑A: Hamilton Anxiety Rating Scale
PHQ-9: Patient Health Questionnaire
BDS-10: Beirut Distress Scale
